# Adherence to the ISHLT Protocol for the Referral of Patients with Idiopathic Pulmonary Fibrosis to the Transplantation Center among of Czech Centers for Interstitial Lung Diseases

**DOI:** 10.1155/2024/5918042

**Published:** 2024-06-30

**Authors:** Martina Sterclova, Martina Doubkova, Lubica Sykorova, Vladimir Bartos, Monika Zurkova, Vladimira Lostakova, Radka Mokosova, Martina Plackova, Ladislav Lacina, Michaela Cimrova, Radka Bittenglova, Pavlina Lisa, Pavla Musilova, Daniel Dolezal, Jana Psikalova, Petra Ovesna, Martina Koziar Vasakova

**Affiliations:** ^1^ Department of Respiratory Medicine 2nd Faculty of Medicine of Charles University and Motol University Hospital, V Uvalu 84, 150 06 Prague, Czech Republic; ^2^ Department of Lung Diseases and Tuberculosis University Hospital Brno, Jihlavska 20, 625 00 Brno, Czech Republic; ^3^ Department of Respiratory Medicine of the University Hospital Hradec Kralove, Sokolska 581, 500 05 Hradec Kralove, Czech Republic; ^4^ Department of Lung Diseases and Tuberculosis University Hospital Olomouc, Zdravotniku 248/7, 779 00 Olomouc, Czech Republic; ^5^ Department of Pulmonary Diseases and Tuberculosis of the Ostrava University Hospital and MF OU, 17. Listopadu 5, 708 00 Ostrava, Czech Republic; ^6^ Department of Pneumology and Phthisiology AGEL Nový Jicin, Purkynova 16, 741 01 Novy Jicin, Czech Republic; ^7^ Department of Respiratory Medicine University Hospital Bulovka, Budinova 67/2, 180 00 Prague, Czech Republic; ^8^ Department of Pneumology and Phthisiology University Hospital Plzen, Edvarda Benese 13, 300 01 Plzen, Czech Republic; ^9^ Pulmonary Department of Jihlava Hospital, Vrchlickeho 4630/59, 586 01 Jihlava, Czech Republic; ^10^ Pulmonary Department Masaryk Hospital Usti nad Labem, Socialni pece 3316/12a, 400 11 Ústí nad Labem, Czech Republic; ^11^ Department of Pneumology and Allergology Kromeriz Hospital, a.s., Havlickova 660 767 01, Kromeriz, Czech Republic; ^12^ Institute of Biostatistics and Analysis Masaryk University Brno, Krenova 72, 602 00 Brno, Czech Republic; ^13^ Department of Respiratory Medicine of the 1st Faculty of Medicine of Charles University and Thomayer University Hospital, Videnska 800, 140 00 Prague, Czech Republic

## Abstract

There are limited data on referral rates and the number of patients with idiopathic pulmonary fibrosis (IPF) who are eligible for lung transplantation. The aim of the present study was to assess adherence to the consensus of the International Society for Heart and Lung Transplantation (ISHLT) for the referral of patients with IPF among Czech interstitial lung disease (ILD) centers. Czech patients who were diagnosed with IPF between 1999 and 2021 (*n* = 1584) and who were less than 65 years old at the time of diagnosis were retrospectively selected from the Czech Republic of the European Multipartner Idiopathic Pulmonary Fibrosis Registry (EMPIRE). Nonsmokers and ex-smokers with a body mass index (BMI) of <32 kg/m^2^ (*n* = 404) were included for further analyses. Patients with a history of cancer <5 years from the time of IPF diagnosis, patients with alcohol abuse, and patients with an accumulation of vascular comorbidities were excluded. The trajectory of individual patients was verified at the relevant ILD center. From the database of transplant patients (1999-12/2021, *n* = 541), all patients who underwent transplantation for pulmonary fibrosis (*n* = 186) were selected, and the diagnosis of IPF was subsequently verified from the patient's medical records (*n* = 67). A total of 304 IPF patients were eligible for lung transplantation. Ninety-six patients were referred to the transplant center, 50% (*n* = 49) of whom were referred for lung transplantation. Thirty percent of potentially eligible patients not referred to the transplant center were considered to have too many comorbidities by the reporting physician, 19% of IPF patients denied lung transplantation, and 17% were not referred due to age. Among Czech patients with IPF, there may be a larger pool of potential lung transplant candidates than has been reported to the transplant center to date.

## 1. Introduction

Despite advances in the treatment of idiopathic pulmonary fibrosis (IPF), it remains a fatal disease. The advent of antifibrotic treatment was suggested to make IPF a chronic disease rather than a fatal disease, but that goal has not been met in many patients [1]. IPF mostly affects patients older than 60 years of age; however, in some patients, the radiological/histological phenotype of usual interstitial pneumonia (UIP) without signs of systemic connective tissue disease and without exposure to organic/inorganic inhalation compounds manifests itself 1-2 decades earlier [2]. The course of IPF is highly heterogeneous: while some patients with IPF have a slow decline in lung function, others may experience rapid progression even after receiving antifibrotic treatment [3].

Early diagnosis is key to treatment success. To improve the access of IPF patients to interstitial lung disease (ILD) centers, a network of 13 centers for diagnosis and treatment was established in the Czech Republic in 2011. The establishment of ILD centers and centralized care for IPF patients allowed one center to create a database in which data from Czech IPF patients have been prospectively collected since 2012. The database evolved into the European Multipartner Idiopathic Pulmonary Fibrosis Registry (EMPIRE) in 2014. Data from IPF patients were also retrospectively entered into the database to evaluate as much information about the disease and its impact on patient survival as possible.

These centers are responsible for the diagnosis and management of IPF, including timely referral to the transplant center at Motol University Hospital, Prague. The Motol transplant center has been providing lung transplantation to Czech patients since 1998, when the first consensus document encompassing lung transplantation indications, contraindications, referrals, and listing criteria was published. Since then, the consensus document has been updated several times, with the most recent update being published in 2021; furthermore, it has been adopted by the Czech transplant center in Motol since its first publication [4–7].

In addition to demographic data from IPF patients, the EMPIRE provides information about their lung functions or comorbidities and information on therapy, including referral to the transplant center. However, we had no information on whether all IPF patients who were potentially eligible for lung transplantation were referred to the transplant center and offered lung transplantation. Recognizing the expected gap should result in closer cooperation between the ILD centers and the transplant center.

In the present study, our objective was to assess adherence to the International Society of Heart and Lung Transplantation (ISHLT) guidelines among Czech ILD centers and to determine whether IPF patients in the Czech Republic have sufficient access to this treatment method.

## 2. Methods

Patients who were diagnosed with IPF in the Czech Republic between 1999 and 2021 and who were less than 65 years old at the time of diagnosis were retrospectively selected from the EMPIRE. Nonsmokers and ex-smokers with a body mass index (BMI) of <32 kg/m^2^ were included in further analyses. Patients with a history of cancer <5 years from the diagnosis of IPF, patients with alcohol abuse, and patients with vascular comorbidities (ischemic heart disease with a history of aortocoronary bypass or percutaneous coronary intervention and/or ischemic stroke or transient ischemic attack and/or peripheral ischemic vascular disease with a history of percutaneous transluminal angioplasty or venous or prosthetic bypass) were excluded. As data on renal function (glomerular filtration) are not mandatory for the use of EMPIRE, renal failure was also used as an exclusion criterion. The criteria used for the selection of lung transplant candidates after full pretransplant assessment followed the guidelines published in 1998, 2006, 2014, and 2021 [4–7].

**Figure 1:**
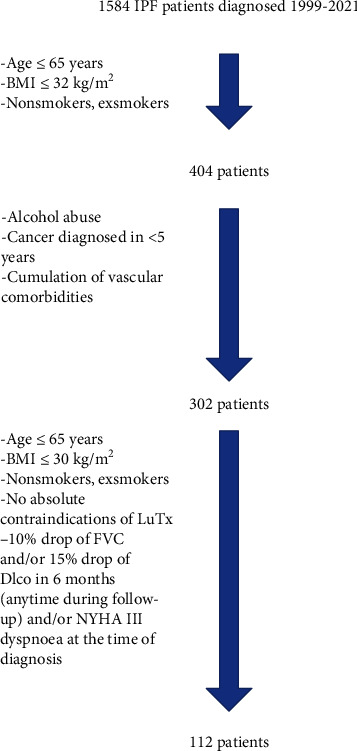
Selection of potential lung transplantation candidates based on EMPIRE data. BMI: body mass index; FVC: forced vital capacity; DLco: diffusing capacity for carbon monoxide; LuTx: lung transplantation; NYHA: New York Heart Association.

Subsequently, ILD centers were surveyed about the outcome of the included patients (referred to the transplant center yes/no (with reasons)). The patient selection process is summarized in Figure 1. From the database of transplant patients (1999-2021, *n* = 541), all transplanted patients with pulmonary fibrosis (*n* = 186) were selected, and the diagnosis of IPF was subsequently verified from the patient's medical records (*n* = 67).

Descriptive statistics were used. Demographic data are expressed as the mean ± standard deviation and median (minimum-maximum value).

Since the establishment of the EMPIRE, ILD centers have been regularly asked to add missing data from the included patients, and the data used for analysis were available for all included subjects.

This manuscript was written in accordance with the STROBE checklist.

## 3. Results

**Figure 2:**
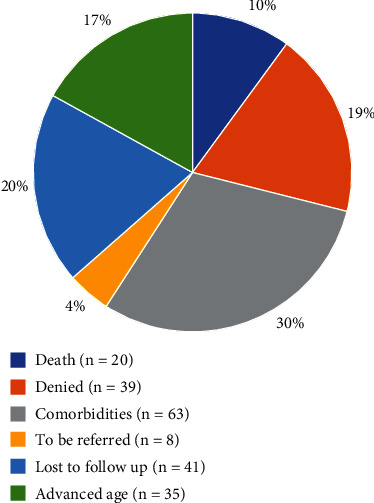
Reasons for not referring potential candidates for lung transplantation to the transplant center (stated by the ILD centers) (*n* = 208). ILD: interstitial lung diseases.

The demographic data of the patients are shown in Table 1.  Table 2 summarizes the data obtained from the EMPIRE, the ILD centers, and the transplant center. By the time the EMPIRE was launched in 2012, 15 IPF patients had undergone transplantation. The reasons why 208 potential candidates for lung transplantation (based on EMPIRE data) were not referred to the transplant center (according to ILD centers) are shown in Figure 2.

**Figure 3:**
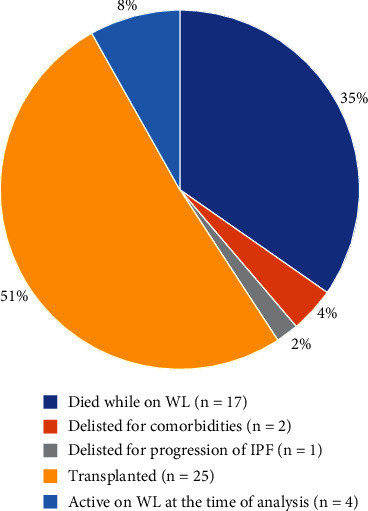
Outcome of ILD patients listed for lung transplantation (*n* = 49). ILD: interstitial lung diseases; WL: waiting list; IPF: idiopathic pulmonary fibrosis.

**Figure 4:**
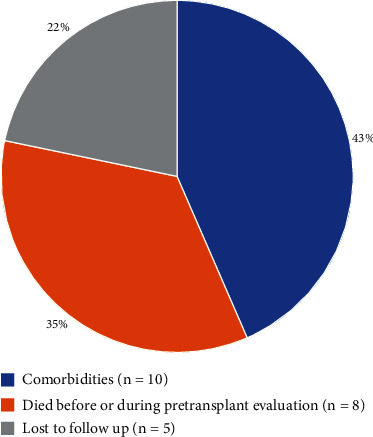
Reasons for not including potential IPF candidates for lung transplantation (based on pretransplant evaluation at the transplant center and discussion with the transplant team).

Of the 96 patients referred, 51% (*n* = 49) were listed for lung transplantation, 24% (*n* = 23) were not listed, and 25% of the patients referred (*n* = 24) were considered to be potential lung transplant candidates at the transplant center at the end of the analysis period.  Figure 3 shows the outcomes of the patients listed, and Figure 4 documents the outcomes of the patients not listed for lung transplantation.

## 4. Discussion

The present study is aimed at assessing adherence to ISHLT guidelines and consensus documents among Czech ILD centers and evaluating whether Czech IPF patients have adequate access to lung transplantation. We found that two-thirds of potential lung transplant candidates were never referred to the transplant center (Figure 2). Data from the EMPIRE showed that of the 1632 patients diagnosed with IPF, 6% were referred, and 19% should possibly be referred to the transplant center. The main reason for nonreferrals was comorbidities (according to the assessment by the ILD center, neither the transplant center nor consecutive ISHLT documents) (30%), followed by a decline (19%). Fifty-one percent of the IPF patients referred were listed for lung transplantation, and 26% underwent transplantation (Figure 3). Twenty-seven patients (19%) were not referred due to their age, which was assumed to be too high by the ILD center specialist. Attention should be given to patients who died on the waiting list or during the pretransplant examination (*n* = 25) (Figure 4).

The existence of the EMPIRE allows us to gain more precise insight into the number of patients who could benefit from lung transplantation, and the present study clearly showed a gap between those who met the referral criteria and those who were referred. We were able to recognize the modifiable factors that had prevented the ILD centers from referring some of the patients. Because the data from the EMPIRE were evaluated at each ILD center, the cooperation between the transplant center and the ILD centers became tighter. Based on these data, a program with feedback on referred patients was introduced, and regular online meetings with referring physicians were scheduled.

This study has several limitations. The EMPIRE is a real-life registry, and as such, it is susceptible to human errors (e.g., incomplete data and missed entries). The number of lung transplantations according to the EMPIRE (*n* = 25) represents only 37% of the actual lung transplantations for IPF (*n* = 67) documented by the transplant center. Interestingly, all 67 transplanted patients were referred to ILD centers. We assume that the discrepancy between the EMPIRE and transplant center data might be explained by the fact that, despite increased awareness of IPF, there are still patients who were referred to ILD centers at advanced disease stages. In this scenario, the patient might have been referred directly to the transplant center without many investigations and without registry entry.

The guidelines regarding the selection of eligible patients changed several times, as did the listing criteria for IPF. The number of transplanted patients (ISHL 2001-2009, *n* = 21, 806; 2010-2018, *n* = 33, 891) and the proportion of IPF patients among transplanted patients (2001-2009, 22.3%; 2010-2018, 29%) also increased over time [8]. With increasing experience, the listing guidelines are becoming stricter, and referral recommendations are becoming more open. Patients with IPF who met the 2021 list criteria would have also met the criteria in 1998. However, patients who met the criteria in 1998 may not have met the criteria in 2021.

There are limited data on IPF patient referral rates to transplant centers. The ISHLT document is straightforward, but the opinions of ILD specialists on which and when IPF patients should be transferred differ [4]. Although patients have the right to be informed about all treatment options, a very strong argument about the feasibility of such an approach in a very common scenario of limited resources speaks against this practice [9, 10]. Discrepancies between guidelines and clinical practice were shown in the study of Swaminathan et al., who reported that 30.4% of IPF patients in the IPF-PRO registry were eligible for lung transplantation (age criterion < 70 years). When reviewing the group of transplanted IPF patients, they observed that only 53.1% of transplanted IPF patients met the eligibility criteria [11].

According to the EMPIRE, 51% of patients referred for IPF were referred for lung transplantation, and 26% were referred for lung transplantation. This ratio is much higher than that in the previously published article by King et al. (8.8% listed and/or transplanted) [12]. The eligibility for lung transplantation in the study mentioned by King et al. was defined by age less than 75 years and BMI less than 35 kg/m^2^.

The trend of accepting candidates with IPF over 65 years of age dominates, especially in the USA [13]. However, because antifibrotic drugs prolong the survival of patients with IPF, the number of patients who are diagnosed after the age of 60 and who, due to antifibrotic treatment, have reached the referral criteria beyond the recommended upper age limit of 65 years has increased over time. Even European centers respond to this fact by increasing the upper limit to accept a candidate with IPF on the waiting list to 67-70 years; therefore, consultation in a transplant center should also be considered for selected patients over 65 years of age [14].

The relatively high number of those listed/transplanted from the group of referred patients (51% vs. 26%) could be explained by the preselection of potential candidates in ILD centers. The fact that 96 of the 304 potentially eligible patients were referred supports this hypothesis. The reason for not being sent to a transplant center was comorbidities (claimed by the ILD center) in 23% of the potential candidates. At least one comorbid condition affects 89% of IPF patients, and multimorbidity, especially in older IPF patients, is a common problem [15]. The prevalence of cardiovascular diseases in patients with IPF listed in the EMPIRE reaches 74%, most frequently represented by coronary heart disease in 23% of patients. Surprisingly, the incidence of lung cancer in EMPIRE patients does not exceed 3.6% [16]. We believe that in the absence of evident absolute contraindications for lung transplantation, it is advisable to consult the patient with the transplant center to avoid withholding this treatment for patients with modifiable barriers to lung transplantation.

All patients who died on the waiting list or during pretransplant evaluation were reviewed closely. In some of them, the cause of death was an unpredictable acute exacerbation of the disease, but some of the patients probably died from advanced disease and a longer waiting list, especially at the beginning of the transplant program. Transplantation of a patient due to an acute exacerbation of IPF is not recommended unless the patient has already been thoroughly examined and optimally placed on a waiting list [17]. However, even for included candidates, it is necessary to respect the decision of the transplant team about the indication of bridging for a specific candidate and choose the scope of intensive care provided accordingly.

The mortality of IPF patients on the waiting list is undoubtedly higher than the currently reported 11% [18, 19], but when evaluating this parameter, it is necessary to consider the fact that the Czech transplant center became a high-volume center with >30 transplants per year only in 2014 [20]. Moreover, the Czech Republic is not included in Eurotransplant, and the donor pool is limited to the Czech Republic and Slovakia.

Figure 1 shows that one of the selection criteria that defines potential lung transplant candidates in the present study was grade III dyspnea (scaled according to the New York Heart Association (NYHA)). The quality of life among potential IPF lung transplant candidates is not mentioned in the 2021 ISHLT consensus, although it is recommended that other diseases that can be treated with lung transplantation be evaluated. The NYHA grade is in good agreement with the quality of life of patients with IPF and the distance that the patient walks in six minutes [21, 22]. According to recent research, the patient's perception of quality of life and the results of objective examinations do not correlate in approximately one-third of patients [23]. The authors of the research justify this discrepancy by the probable influence of the so-called social determinants of health, i.e., the conditions in which the patient was born, grew up, lived, worked, and aged [24]. In 20% of the patients included in the research mentioned above, the degree of symptoms was more pronounced than the objectively assessed severity of the disease, and in all cases, they were persons with a complicated psychosocial profile. However, considering the limited survival after lung transplantation and the unpredictable outcomes, our transplant center only lists those with pronounced dyspnea and poor quality of life due to dyspnea in our transplant center.

There are more IPF patients who could be offered lung transplantation than we met at the transplant center. We believe that closer cooperation between the transplant center and the ILD centers would increase awareness of this therapy and thus provide a better perspective for eligible IPF patients. We have improved the feedback for ILD centers in the form of regular online meetings, where we discuss the latest referrals and their outcomes.

## 5. Conclusions

We suggest that there may be a larger pool of potential lung transplant candidates among Czech patients with IPF than has been reported to date. Closer cooperation with referring ILD centers could offer lung transplantation and thus improve the perspective of more eligible IPF patients.

## Figures and Tables

**Table 1:** Demographic data of the study population.

Group (*N*)	IPF total (1584)	^†^Selection 1 (404)	^‡^Selection 2 (302)	^§^Potentially transplantable (112)	Transplanted (67)	*p* value^1^
Age (years, mean ± SD)	69.9 ± 8.6^a^	58.6 ± 5.5^b^	58.8 ± 5.5^b^	57.2 ± 6.6^b^	58.2 ± 8.2^b^	<0.001
Gender (M/F)	1155/429	290/114	229/73	76/36	52/15	0.446
FVC (% ev, mean ± SD)	73 ± 18.7^a^	73 ± 18.7^a^	73 ± 18.7^a^	70.7 ± 19.7^a^	51.5 ± 12.0^b^	<0.001
Comorbidities (*n* ± SD)	4.2 ± 2.5	3.8 ± 2.6	3.5 ± 2.4	3.1 ± 2.1	1.9 ± 2.2	
Comorbidities (med (min–max))	4 (0-14)^a^	3 (0-14)^b^	3 (0-12)^b^	3 (0-9)^b^	2 (0-5)^c^	<0.001

^†,‡,§^Refer to Figure 1. ^1^*p* value from ANOVA, Fisher's exact test, or Kruskal-Wallis test. ^a,b,c^Homogeneous groups from post hoc pairwise comparisons (cells sharing the same letter are not significantly different at the 5% level). IPF: idiopathic pulmonary fibrosis; SD: standard deviation; M: male; F: female; FVC: forced vital capacity; ev: expected value.

**Table 2:** Comparison of data from the EMPIRE, the ILD centers, and the transplant center.

	EMPIRE	ILD centers	Transplant center
Referable (EMPIRE)/referred (ILD centers) (*n*)	302	96	NA
Transplantable (EMPIRE)/transplanted (ILD centers)/transplanted (transplant center database) (*n*)	112	25	67

ILD: interstitial lung disease; Tx: transplantation.

## Data Availability

Original data are available upon reasonable request.
